# Evaluation of Immunotropic Activity of Iridoid-Anthocyanin Extract of Honeysuckle Berries (*Lonicera caerulea* L.) in the Course of Experimental Trichinellosis in Mice

**DOI:** 10.3390/molecules27061949

**Published:** 2022-03-17

**Authors:** Jolanta Piekarska, Marianna Szczypka, Michał Gorczykowski, Anna Sokół-Łętowska, Alicja Z. Kucharska

**Affiliations:** 1 Division of Parasitology, Department of Internal Medicine and Clinic of Horses, Dogs and Cats, Wrocław University of Environmental and Life Sciences, Norwida 31, 50-375 Wrocław, Poland; michal.gorczykowski@upwr.edu.pl; 2 Department of Pharmacology and Toxicology, Wrocław University of Environmental and Life Sciences, Norwida 31, 50-375 Wrocław, Poland; marianna.szczypka@upwr.edu.pl; 3 Department of Fruit, Vegetable and Plant Nutraceutical Technology, Wrocław University of Environmental and Life Sciences, Chełmońskiego 37, 51-630 Wrocław, Poland; anna.sokol-letowska@upwr.edu.pl (A.S.-Ł.); alicja.kucharska@upwr.edu.pl (A.Z.K.)

**Keywords:** *Lonicera caerulea* L., *T. spiralis*, immunomodulation

## Abstract

Our experiment determined the immunotropic activity of a natural, iridoid-anthocyanin extract from honeysuckle berry (*Lonicera caerulea* L.) (LC). The extract was administered to mice infected with *Trichinella spiralis*, orally at a dose of 2 g/kg bw, six times at 24 h intervals (from day 3 prior to the infection to day 3 post-infection (dpi) with *T. spiralis*. At 5, 7, 14, and 21 dpi, samples of blood, spleen, and mesenteric lymph nodes (MLN) were collected, and isolated lymphocytes were analyzed by flow cytometry. The splenocyte proliferation was estimated with MTT testing, and the intensity of intestinal and muscle infection was also studied. LC stimulated the local immune system by inducing lymphocyte proliferation in the spleen 7 dpi and altered the percentage and absolute count of B (CD19^+^) and T (CD3^+^, CD8^+^) cells 7, 14, and 21 dpi in the peripheral blood. LC extract affected the dynamics of expulsion of adult *Trichinella* from the intestines and prolonged the intestinal phase of the infection but did not change the number of larvae in the muscles. These results suggest that *Lonicera caerulea* L. fruit extract modulates murine cellular immune response during intestinal phase of *T. spiralis* infection but shows no antiparasitic activity.

## 1. Introduction

Trichinellosis, caused by the nematode *Trichinella spiralis,* is a worldwide zoonotic infection that causes significant economic loss in pork production and has a notable impact on human health. The source of human and animal infections is uncooked meat that contains encysted *T. spiralis* larvae. The entire life cycle of *Trichinella* is completed in a single host, where the parasite can steadily reside for several years. The presence of adult *Trichinella* in the host intestines and larval forms in the muscles induces a complex immune response, in which effector mechanisms are controlled by a subset of T lymphocytes, CD4^+^ cells, which can polarize into Th1- or Th2-response upon an antigenic stimulation [1,2]. In the intestinal phase of *T. spiralis* infection, a short, Th1-type immune response predominates. Then Th2-type, involving the production of cytokines such as IL-4, IL-5, and IFN-γ and prominent mucosal mastocytosis, contributes to parasite expulsion [3,4]. Moreover, Th17 cells are induced to produce a proinflammatory cytokine IL-17, which plays a role in intestinal inflammation. During the muscle (chronic) stage of the infection, the stable Th2 immune response is maintained, which may help in alleviating tissue damage and strengthening tissue repair. Treg cells are also induced in the muscle phase to produce regulatory and anti-inflammatory cytokines that reduce the host immune response [5]. The composition and activation of immune cells can be regulated by factors of parasitic origin.

Honeysuckle berries (*Lonicera caerulea* L. var. *kamtschatica* Sevast) (LC) have been appreciated for centuries in the folk medicine of northern Russia, China and Japan, and recently also in Europe and North America as a source of numerous bioactive compounds with health-promoting properties [6]. High biological activity of phenolic acids, flavanols, flavones, isoflavones, and anthocyanins, but also iridoids from LC fruit was confirmed in numerous in vitro and in vivo studies conducted in many laboratories worldwide [7,8]. For example, anticancer properties of purified anthocyanin extract from LC berries were demonstrated in human and murine cell lines (SMMC-7721 and H22) [9]. Polyphenols (including anthocyanins) from LC fruit can modulate the intestinal environment and alleviate liver injury by suppressing oxidative stress-related pathways and altering the composition of the intestinal microbiota. The compounds exert strong antioxidant and anti-inflammatory effects that may be helpful in wound healing [10,11,12,13]. LC fruit extract administered to mice and rats with disorders of lipid and glucose metabolism showed hepatoprotective and anti-inflammatory effects [10,14,15]. Polyphenols from LC berries affected intestinal microflora in mice fed with a high-fat diet. In addition, supplementation of mice with LC fruit extract reduced serum levels of IL-2, IL-6, MCP-1, and TNF-α, as well as endotoxin levels in both serum and the liver [15,16].

The above-mentioned findings encouraged further studies on the effects of plant anthocyanins and iridoids on the immune response in the course of experimental trichinellosis in mice. Our previous work reported a modulatory effect of iridoid-anthocyanin extract of *Cornus mas* L. on the course of experimental trichinellosis in mice [17]. Both extracts, from *Cornus mas* L. and LC fruit, are characterized by high content of iridoid and polyphenolic compounds in different proportions. Biological activity of plant preparations is a result of the relative content of many active substances that differ depending on the plant species.

The aim of our study was to find out how the administration of iridoid-anthocyanin extract of LC fruit affects lymphocyte subsets in the blood, spleen, and mesenteric lymph nodes (MLN) and blood parameters in *T. spiralis* infected mice, and how the extract may modulate the intestinal and muscular phase of the infection.

## 2. Results

### 2.1. Flow Cytometry Analysis

#### 2.1.1. Effects of In Vivo Administration of LC on the Subpopulations of MLN Splenocytes and Lymphocytes

LC administration did not change the percentage and absolute count of T splenocytes (CD3^+^, CD4^+^ and CD8^+^) and B splenocytes (CD19^+^) in *T. spiralis*-infected mice (Group T + LC) (Table 1 and Table 2).

In uninfected mice treated with LC (Group LC), a decrease in the percentage of B splenocytes was observed 5 (*p* < 0.01), 7, and 14 dpi (*p* < 0.05). This was accompanied by an increase in the percentage of T splenocytes: CD4^+^ cells on day 5, and CD8^+^ cells on days 7 and 14 (*p* < 0.05) (Table 1). However, no changes in the absolute splenocyte count were noticed (Table 2).

The CD4^+^/CD8^+^ splenocyte ratio was not affected after LC administration either in *T. spiralis*-infected or uninfected mice (Table 1).

Administration of LC did not affect the percentage of lymphocytes in the MLN of *T. spiralis*-infected mice (Table 3). At 7 dpi, an increase in the absolute count of B mesenteric lymph node cells (CD19^+^) was observed in LC-treated infected mice (*p* < 0.05) (Table 4).

In uninfected mice, LC administration decreased the percentage and absolute count of CD4^+^ mesenteric lymph node cells 5 dpi (*p* < 0.01). It resulted in a drop of CD4^+^/CD8^+^ ratio (*p* < 0.05). On day 14, LC lowered the percentage of B mesenteric lymph node cells (*p* < 0.05) (Table 3 and Table 4).

#### 2.1.2. Effects of In Vivo Administration of LC on the Subsets of T and B Lymphocytes in Peripheral Blood

Peripheral blood of the mice infected with *T. spiralis* (Group T + LC) and receiving LC had higher percentage of CD8^+^ T lymphocytes (*p* < 0.05) 14 dpi, and lower percentage of CD19^+^ B cells (*p* < 0.05) 21 dpi (Table 5). At the same time, a drop in the absolute count of B cells (*p* < 0.01) was noticed (Table 6). In this group, a decrease in the absolute count of CD8^+^ cells was observed on days 7 and 21 post-infection. This was accompanied by a significant increase in the absolute count of CD3^+^ lymphocytes (*p* < 0.05) 14 dpi, followed by its decrease 21 dpi (*p* < 0.01) (Table 6).

In uninfected mice, the iridoid-anthocyanin preparation from LC fruit reduced the percentage and absolute count of CD3^+^ (*p* < 0.01) and CD8^+^ cells (*p* < 0.05) in the blood 7 dpi. The parameters then rose on day 21 (*p* < 0.01). Simultaneously, the percentage of B (CD19^+^) lymphocytes in peripheral blood was boosted on day 7 (*p* < 0.01) and reduced on day 21 (*p* < 0.05). This effect did not correlate with changes in the absolute count of these cells (Table 5 and Table 6). In addition, an elevated CD4^+^/CD8^+^ ratio (*p* < 0.05) was detected 5 dpi (Table 5) and a decrease in the absolute number of CD4^+^ lymphocytes on day 7 and an increase in this subpopulation on day 21 was observed (Table 6).

### 2.2. Effects of Aqueous Extracts of Lonicera caerulea L. Fruit on Lymphocyte Proliferation

LC administration enhanced the proliferation of splenocytes in the mice infected with *T. spiralis* on day 7 (*p* < 0.05) in the presence of 0.9 µg/mL ConA. In uninfected animals treated with LC, the proliferation index at all time points was not significantly different compared to the control animals (Table 7).

### 2.3. Effects of Aqueous Extracts of Lonicera caerulea L. Fruit on the Hematological Parameters

LC preparation significantly lowered the values registered for white and red blood cells 7 and 21 dpi in *T. spiralis*-infected mice. On day 7, a decrease was noticed in white blood cell fraction (MID) and erythrocyte size differentiation indices MCH and RDW (-SD; -CD).

At 21 dpi, there was a drop in WBC and lymphocyte (LIMF) count and a reduction in RBC count and HGB concentration accompanied by an increase in MCV index (Table 8).

In uninfected mice, the preparation significantly affected hematological parameters on all test dates. On day 5, there was a decrease in the percentage of lymphocytes (LYM%) and MCHC, and an increase in granulocytes (GRAN%). On day 7, a decline in WBC, LIMF, GRAN, RBC count, HGB concentration, and HCT values was observed. On day 14, lowered percentage and absolute value of GRAN was seen with a boost in red blood cell indices (RBC, HGB and HCT), and an increase in the percentage of lymphocytes. On day 21, a decrease in the percentage of lymphocyte and a significant gain in the percentage of granulocyte and platelet parameters (PLT and PCT) were observed (Table 9).

### 2.4. Parasite Burden—The Number of Adults and Muscle Larvae

LC preparation did not significantly affect the number of adult parasites in the intestines 5 and 7 dpi, or muscle larvae 60 dpi. The expulsion of *T. spiralis* from the intestines of the mice receiving LC was slower than in the control group. The preparation prolonged the intestinal phase of trichinellosis (Table 10).

## 3. Discussion

Plants are a valuable and cheap source of compounds with diverse biological activities. They are increasingly important in the prevention and treatment of diseases, including those caused by parasites. There are many studies confirming both immunomodulatory [18,19] and antiparasitic [20,21] properties of plant active substances from the group of anthocyanins and iridoids. LC is an excellent source of iridoids, which are rarely present in other fruit. The most abundant compounds in LC berries are anthocyanins with predominance of cyanidin-3-*O*-glucoside [8]. Results of recent studies indicated potential biological activity of LC, especially its antioxidant, anti-inflammatory, neuroprotective, cardioprotective, and antidiabetic effects [22]. Many studies examined the antimicrobial activity of LC. An in vitro study demonstrated that LC extract inhibited growth of *Streptococcus pyogenes* and suppressed biofilm formation in a dose-dependent manner [23]. Molina et al. [24] reported that *Lonicera caerulea* L. berry extract had bactericidal capacity against all tested bacterial strains, both Gram-positive *Bacillus cereus, Staphylococcus aureus,* and *Listeria monocytogenes*; and Gram-negative *Escherichia coli*, *Enterobacter cloacae*, and *Salmonella typhimurium*. Regarding antifungal properties, the strongest effect of LC extract was demonstrated on *Aspergillus versicolor* and *Trichoderma viride*. The effect of LC extract on the course of parasitic infections has not yet been studied.

T lymphocytes play an essential role in both early host response and subsequent pathogenesis of organ lesions in the course of *T. spiralis* infection. Activation of a specific subpopulation of T cells (Th1 or Th2) and the profile of cytokines secreted by them determine the activation of cellular or humoral response.

Iridoid-anthocyanin aqueous extract of *Cornus mas* fruit (CM) was shown to stimulate murine immune response during *T. spiralis* infection. Apart from affecting hematological parameters and proliferative activity of the lymphocytes, CM altered the percentage and absolute number of T cell subpopulations and B lymphocytes in the spleen and MLN of *T. spiralis* infected mice, and finally contributed to a decrease in intestinal parasite burden [17].

In the present study, LC extract exerted only slight and transient effects on the subsets of lymphocytes from the spleen and MLN. In the mice infected with *T. spiralis*, administration of LC only selectively altered the percentage and absolute count of B (CD19^+^) and T (CD3^+^ and CD8^+^) lymphocytes in peripheral blood and absolute count of B (CD19^+^) cells in MLN, but it did not influence the percentage and absolute number of splenocytes. Moreover, significant changes in the proliferation index of Con A-stimulated lymphocytes were observed in the spleen of *T. spiralis* infected mice. The mechanism of LC action may result from the specific composition of active substances, especially high level of polyphenolic compounds and their biological properties. Some researchers showed an immunomodulatory effect of LC, including the effect on lymphocyte activity and cytokine production. The major bioactive anthocyanin of LC, cyanidin-3-*O*-glucoside, downregulated Th2 cytokine synthesis (IL-4, IL-13), but it did not affect Th1 cytokine production (IL-2, IFN-γ, IL-12). In the present study, such a mechanism of action of cyanidin-3-*O*-glucoside from LC extract could explain slower expulsion of *Trichinella* and prolongation of the intestinal phase of the infection [25,26]. Minami et al. [27] reported that *Lonicera* *caerulea* var. *emphyllocalyx* extract (LCEE) in *Streptococcus* *pyogenes*-infected mice suppressed the production of proinflammatory cytokines TNF-*α* and IFN-γ by splenocytes and MLN cells. Wu et al. [12,16,28] showed that polyphenols from LC berry reduced the serum levels of cytokines (IL-2, IL-6, TNF-α, monocyte chemotactic protein-1—MCP-1) in such experimentally induced pathologies as mice fed with high-fat diet, lipopolysaccharide (LPS)-induced mouse paw edema, or a murine model of nonalcoholic steatohepatitis. A similar inhibitory effect of LC on the production of cytokine (IL-1β, IL-6 TNF-α) and nitric oxide (NO) serving as an inflammatory marker was noticed [11,29,30]. Interestingly, as confirmed in vitro and in vivo, the anti-inflammatory properties of LC may be related to inhibiting the nuclear factor kappa B (NF-κB)-dependent signaling pathway and the subsequent production of proinflammatory mediators [10]. However, some anthocyanins (cyanidin-3-glucoside, cyanidin-3-rutinoside, and chlorogenic acid) showed no or weak inhibitory effects on the level of inflammatory mediators and the expression of inducible NO synthase and cyclooxygenase-2 (COX-2) [10]. NF-κB controls the expression of genes that regulate many important cellular processes, such as cell growth and proliferation, apoptosis or immune reactions, and cell response to stress caused by various factors (including helminth infection). Regulation of inflammatory cytokine induction via NF-κB pathways was demonstrated as an important mechanism in parasite infection [31]. The excretory-secretory (ES) products of helminths can inhibit activation of NF-κB that regulates LPS-stimulated pro-inflammatory response in macrophages [4]. Wang et al. [32] reported that LC berry extract may also affect immune cells via inhibition of LPS-induced expression of Toll-like receptors (TLRs) TLR2 and TLR4. In the course of *T. spiralis* infection TLRs may regulate the response of dendritic cells (DCs) and macrophages, as well as the other immune cells of the innate immune system such as mast cells [33]. ES products of *T. spiralis* play an important role in the modulation of the host immune response, and at different stages of *T. spiralis* infection may regulate the expression of TLR2 and TLR4 on macrophages and NF-κB signaling pathways [4].

Modulatory effects of *Lonicera caerulea* L. polyphenols (LCP) on the intestinal environment was also demonstrated. Results of this study confirmed that LCP can alleviate high-fat diet-induced intestinal oxidative stress and intestinal inflammation by significantly downregulating the expression levels of pro-inflammatory factors. LCP also affected intestinal functions via regulating intestinal microbiota and intestinal cytokines [13].

This study showed that in the course of experimentally induced trichinellosis in mice, aqueous extract of *Lonicera caerulea* L. affected proliferative activity of splenocytes, altered the percentage and absolute count of B (CD19^+^) and T (CD3^+^, CD8^+^) cells in peripheral blood, and modified selected hematological parameters.

Although the immunotropic activity of LC did not expressly change the intensity of *T. spiralis* infection, its immunomodulatory properties could be used to mitigate the intestinal inflammation associated with trichinellosis, which of course requires further research.

## 4. Materials and Methods

### 4.1. Plant Material

Honeysuckle berries (*Lonicera caerulea* L. var. *kamtschatica* Sevast.) were obtained as a mixture of cultivars (“Zojka”, “Wojtek”, “Jolanta”) from plantations near Skierniewice (51°57′00.4″ N 20°10′56.0″ E), (Poland).

#### Chemicals

Acetonitrile, formic acid, and methanol were purchased from Sigma-Aldrich (Steinheim, Germany). Standards of cyanidin 3-*O*-glucoside (C 3-glc) and loganic acid were purchased from Extrasynthese (Genay, France), and quercetin 3-*O*-glucoside and 5-*O*-caffeoylquinic acid were purchased from Sigma-Aldrich (Steinheim, Germany).

#### Extraction of Anthocyanins and Iridoids

Juice extracted from frozen ripe honeysuckle berries (LC) was purified by removing sugars and organic acids on Amberlite XAD-16 resin column (Rohm and Haas, Chauny Cedex, France). Phenolic compounds were eluted with 80% ethanol. The eluate was concentrated under vacuum at 40 °C using Rotavapor (Unipan, Warsaw, Poland). The HB extract obtained by freeze-drying was analyzed by UPLC-qTOF-MS/MS (Waters Corp., Milford, MA, USA) and HPLC-PDA (Dionex, Germering, Germany). The extraction yield from the plant material was 0.4%. The dry matter content in the preparation was 96%.

#### Identification of Iridoids and Polyphenols by UPLC-qTOF-MS/MS

The method was previously described by Kucharska et al. [8]. The honeysuckle extract was dissolved in 50% aqueous methanol containing 0.1% HCl and filtered through a 0.22 µm filter. Identification of compounds was performed using the Acquity ultra-performance liquid chromatography (UPLC) system coupled with a quadrupole-time of flight (Q-TOF) MS instrument (UPLC/Synapt Q-TOF MS, Waters Corp., Milford, MA, USA) with an electrospray ionization (ESI) source. Separation was achieved on the Acquity BEH C18 column (100 mm × 2.1 mm i.d., 1.7 μm; Waters). The mobile phase was a mixture of 2.0% aq. formic acid (A) (*v*/*v*) and acetonitrile (B). The gradient program was as follows: initial conditions—1% B in A, 0–12 min—25% B in A, 12–12.5 min—100% B, 12.5–13.5 min—1% B in A. The flow rate was 0.45 mL/min, and the injection volume was 5 μL. The column was operated at 30 °C. UV–Vis absorption spectra were recorded online during UPLC analysis, and the spectral measurements were made in the wavelength range of 200–600 nm, in steps of 2 nm. The major operating parameters for the Q-TOF MS were as follows: capillary voltage 2.0 kV; cone voltage 40 V; cone gas flow of 11 L/h; collision energy 28–30 eV; source temperature 100 °C; desolvation temperature 250 °C; collision gas, argon; desolvation gas (nitrogen) flow rate, 600 L/h; data acquisition range, *m/z* 100–2000 Da; ionization mode, negative and positive. The data were collected with Mass-LynxTM V 4.1 software (Waters Corp., Milford, MA, USA) (Table 11).

#### Quantification of Iridoids and Polyphenols by HPLC-PDA

The procedure was previously described by Sokół-Łętowska et al. [34]. The honeysuckle extract was dissolved in 50% aqueous methanol containing 0.1% HCl and filtered through a 0.45 µm filter. The HPLC-PDA analysis was performed using a Dionex (Germering, Germany) system equipped with a diode array detector model Ultimate 3000, a quaternary pump LPG-3400A, an autosampler EWPS-3000SI, a thermostatted column compartment TCC-3000SD, and controlled by Chromeleon v.7.2 software (Thermo Scientific Dionex, Sunnyvale, CA, USA). The Cadenza Imtakt column CD-C18 (75 × 4.6 mm, 5 μm) with a guard column was used. The mobile phase was composed of a solvent C (4.5% aq. formic acid, *v*/*v*) and a solvent D (100% acetonitrile). The elution system was as follows: 0–1 min 5% D in C, 1–20 min 25% D in C, 20–21 min 100% D, 21–26 min 100% D, 26–30 min 5% D in C. The flow rate of the mobile phase was 1.0 mL/min, and the injection volume was 20 μL. The column was operated at 30 °C. The runs were monitored at the following wavelengths: iridoids at 245 nm, phenolic acids at 320 nm, flavonols at 360 nm, and anthocyanins at 520 nm. The content of iridoids was expressed as loganic acid, anthocyanins as cyanidin 3-*O*-glucoside, flavonols as quercetin 3-*O*-glucoside, and phenolic acids as 5-*O*-caffeoylquinic (chlorogenic) acid equivalents. The results were expressed as milligrams per 1 g of dry weight (dw) (Table 11).

### 4.2. Animals

The study was carried out in CFW mice (*n* = 108), males and females aged 8–10 weeks, weighing approximately 25–30 g, derived from the Centre for Genetic Engineering at the Faculty of Veterinary Medicine, Wrocław University of Environmental and Life Sciences (No. in the register of breeders/suppliers: 021). The study protocol was approved by the Local Ethical Committee for animal experiments in Wrocław, Poland (Resolution No. 107/2018/P2). The animals were housed in an air-conditioned room (23 ± 2 °C), with a 12 h light/12 h dark cycle, and had unlimited access to food and tap water. The mice were orally infected with 200 *T. spiralis* larvae. All efforts were made during the experiments to minimize animal suffering. 

### 4.3. Parasitological Material

*Trichinella spiralis* isolate (T1, ISS1820, Poland) was maintained by serial passages in CFW inbred mice at the Division of Parasitology, Wrocław Faculty of Veterinary Medicine. The infective larvae were recovered from the muscle tissue of mice infected two to three months before via digestion with 1% pepsin/HCl solution for 1 h at 37 °C.

### 4.4. Administration of Aqueous Extract of Lonicera caerulea L. (LC) Fruit and Experimental Design

The iridoid-anthocyanin preparation from *Lonicera caerulea* L. (LC) fruit was orally administered to mice at a dose of 2 g/kg bw six times at 24 h intervals (from day 3 prior to the infection to day 3 post infection with *T. spiralis*). The preparation was dissolved in 0.2 mL of distilled water and administered orally by a stomach tube to 30 uninfected mice and 30 mice infected with *T. spiralis*. The dose of the preparation was based on the study of Kim et al. [35].

The experimental groups were as follows:

Group T: mice infected with *T. spiralis* larvae (30 individuals)
Group T + LC: mice infected with *T. spiralis* larvae and receiving LC (30 individuals)
Group LC: uninfected mice receiving LC (24 individuals)
Group C: uninfected mice (control) (24 individuals)

#### 4.4.1. Determination of Lymphocyte Subsets from Blood, Spleen and Mesenteric Lymph Nodes (MLN)

At 5, 7, 14, and 21 dpi the mice were anesthetized by inhalation with 2–3% isoflurane (Forane, Aesica Queenborough Limited, Queenborough, UK). Blood samples from the orbital sinus were taken from each mouse after a quick removal of the eyeball from the socket with a pair of tissue forceps and transferred into tubes containing hematological anticoagulant ethylenediaminetetraacetic acid (EDTA) [36]. Then the mice were sacrificed by cervical dislocation, and spleens and mesenteric lymph nodes were removed. Cell suspensions from lymphatic organs were obtained by passing the organs through a nylon mesh into 1 mL of phosphate-buffered saline (PBS, Institute of Immunology and Experimental Therapy, Wrocław, Poland). The isolation of lymphocytes from the blood and spleen and mesenteric cell suspensions was performed as described previously [37]. The splenocytes and MLN lymphocytes in the suspension (1 × 10^7^ cells/mL) were stained with a monoclonal rat anti-mouse CD4:FITC/CD8:RPE dual color reagent (Serotec, Kidlington, UK) or a monoclonal rat anti-mouse CD19:FITC/CD3:RPE dual color reagent (Serotec, Kidlington, UK) at the dilution recommended by the manufacturer. After incubation with monoclonal antibodies at 4 °C for 30 min, the lymphocytes were washed twice with PBS and centrifuged (380 g, 8 min, 4 °C). Fluorescence was measured with a flow cytometer (BD FACSCalibur, BD Biosciences, San Jose, CA, USA). The acquired data were analyzed using CellQuest Pro software. The percentage and total lymphocyte count for CD subsets of CD19^+^, CD3^+^, CD4^+^, and CD8^+^ cells in spleens and MLN as well as CD4^+^/CD8^+^ ratio were determined. The total lymphocyte count was calculated based on the total count of lymphocytes in lymphatic organs from individual animals.

#### 4.4.2. Lymphocyte Proliferation Assay

The spleen lymphocytes were cultured in RPMI 1640 medium (R6504 Sigma-Aldrich, Saint Louis, MO, USA) supplemented with NaHCO_3,_ HEPES 5 mM, sodium pyruvate 1 mM, gentamycin (50 mg/L, Polfa, Tarchomin, Poland) and heat-inactivated fetal bovine serum (10%) (Gibco Limited, No 26010074, Lower Hutt, New Zealand). The cells at a concentration of 4 × 10^5^ cells per 150 µL final volume were plated into 96-well microtiter plate (Costar 3596, Corning Incorporated, Glendale, CA, USA). Concanavalin A (Con A) (Sigma-Aldrich, Saint Louis, MO, USA) at the final working concentrations of 0.9, 0.45 and 0.225 µg/mL was added, and the cells were cultured for 72 h at 37 °C in 5% CO_2_. Colorimetric lymphocyte proliferation was evaluated using MTT (3-(4,5-dimethylthiazol-2-yl)-2,5-diphenyltetrazolium bromide) method. At 3 h before the end of the incubation, 25 µL MTT (5 mg/mL; Sigma-Aldrich, Saint Louis, MO, USA) was added to each well and the plates were further incubated at 37 °C in 5% CO_2_ humidified atmosphere. Then, 125 µL of a lysis buffer (13% SDS, 40% N,N-DMF, pH 4.7) was added, and the entire sample was incubated under the same conditions for the next two hours. Absorbance was then measured at 540 nm against reference wavelength of 620 nm and proliferation index (PI) was determined. The PI is expressed with average optical density (OD) value for mitogen-stimulated cells divided by average OD for the control (non-stimulated cells).

#### 4.4.3. Hematological Analyses

Blood samples were taken 5, 7, 14, and 21 dpi. Six mice from each group were anesthetized by inhalation with 2–3% isoflurane as described above. The following hematological indices were determined in blood samples with EDTA, using a standard hematology analyzer (PE-6800 Procan Electronics Inc., Shenzhen, Guangdong, China): white blood cell (WBC) count subdivided into lymphocytes (LYM), monocytes (MON), and granulocytes (GRAN); different fractions—medium and large leukocytes (MID); red blood cell count (RBC), hemoglobin concentration (HGB); hematocrit value (HCT); red blood cell indices (MCH, MCHC, MCV); platelet indices: platelet count (PLT), platelet volume (MPV), platelet anisocytosis index or platelet volume variation (PDW), plateletcrit or the ratio of platelet volume to total blood volume (PCT), and percentage of large platelets (P-LCR).

As the apparatus differentiated only between three white blood cell populations, manual morphology was performed in blood smears, calculating the percentages and absolute counts of WBC. A total of 200 cells in the blood smear were counted and differentiated to classify individual leukocyte types, such as neutrophils, eosinophils, lymphocytes, and monocytes.

### 4.5. Parasitological Studies (Determination of the Parasite Burden)

Adult parasites were isolated 5, 7, 14, and 21 dpi, and counted by incubation of small intestines in 0.9% NaCl at 37 °C in Baermann funnels overnight. On 60 dpi, the number of muscle larvae was examined by artificial digestion of the entire eviscerated and minced mice carcasses (according to the above-mentioned method).

### 4.6. Statistical Analysis

The data were subjected to t-Student’s test or Mann–Whitney U test to analyze and determine their statistical significance (individually for the groups of infected (T and T + LC) and uninfected (L and C) mice. One-way analysis of variance (ANOVA) and Tukey’s test were used for differences between groups (for the results of proliferation test). *p*-values < 0.05 were considered significant. Results are shown as means ± SD (standard deviation). Calculations were carried out using STATISTICA ver. 13.1 software package.

## Figures and Tables

**Table 1:** Percentage of B splenocytes (CD19^+^) and T splenocytes (CD3^+^, CD4^+^, CD8^+^) in the mice infected with *T. spiralis* (Group T), infected with *T. spiralis* and receiving LC (Group T + LC), uninfected with *T. spiralis* and receiving LC (Group LC), and uninfected mice (control) (Group C). Mean values (*n* = 6) and standard deviations are presented. *p* < 0.05 *, *p* < 0.01 **.

Group	Dpi	%CD19^+^ x ± SD	%CD3^+^ x ± SD	%CD4^+^ x ± SD	%CD8^+^ x ± SD	CD4^+^/8^+^ x ± SD
T	5	68.0 ± 7.2	23.9 ± 6.7	20.8 ± 5.7	5.2 ± 0.7	4.0 ± 0.8
	7	60.3 ± 5.5	30.6 ± 4.7	22.1 ± 4.4	7.2 ± 1.2	3.1 ± 0.7
	14	57.8 ± 4.9	26.5 ± 5.4	19.4 ± 3.7	7.0 ± 1.9	2.8 ± 0.3
	21	49.9 ± 9.0	25.3 ± 6.4	16.2 ± 5.4	4.7 ± 0.9	3.5 ± 1.1
T + LC	5	65.2 ± 1.8	25.6 ± 2.5	21.6 ± 3.4	5.1 ± 1.1	4.3 ± 0.4
	7	57.3 ± 4.9	31.4 ± 6.0	22.6 ± 5.3	7.4 ± 1.2	3.1 ± 0.6
	14	48.0 ± 2.3	30.3 ± 7.2	21.9 ± 8.0	6.0 ± 1.7	3.6 ± 0.3
	21	53.8 ± 10.0	26.4 ± 7.0	18.0 ± 4.5	5.4 ± 2.1	3.5 ± 0.7
LC	5	57.5 ± 3.6 **	32.8 ± 2.1	26.3 ± 1.7 *	6.1 ± 0.4	4.3 ± 0.4
	7	55.7 ± 6.1 *	35.4 ± 5.7	24.8 ± 3.1	10.0 ± 2.2 *	2.6 ± 0.5
	14	48.0 ± 4.2 *	44.9 ± 4.4	33.9 ± 3.2	11.0 ± 2.0 *	3.2 ± 0.7
	21	49.3 ± 5.8	38.8 ± 4.1	24.3 ± 3.9	7.5 ± 0.5	3.3 ± 0.7
C	5	68.4 ± 4.2	24.6 ± 4.1	19.7 ± 2.2	6.3 ± 1.7	3.3 ± 0.9
	7	66.2 ± 2.6	26.8 ± 1.5	21.0 ± 1.4	6.0 ± 2.0	3.8 ± 1.3
	14	59.1 ± 3.4	33.0 ± 4.2	26.4 ± 5.8	5.7 ± 1.1	4.7 ± 1.1
	21	57.3 ± 4.9	33.3 ± 4.4	24.3 ± 3.7	6.0 ± 1.2	4.1 ± 0.8

**Table 2:** Absolute count (cells × 10^6^) of splenocyte B (CD19^+^) and T (CD3^+^, CD4^+^, CD8^+^) subpopulations in the mice infected with *T. spiralis* (Group T), infected with *T. spiralis* and receiving LC (Group T + LC), uninfected with *T. spiralis* and receiving LC (Group LC), and uninfected mice (control) (Group C). Mean values (*n* = 6) and standard deviations are presented.

Group	Dpi	CD19^+^ x ± SD	CD3^+^ x ± SD	CD4^+^ x ± SD	CD8^+^ x ± SD
T	5	16.90 ± 3.36	6.23 ± 2.92	5.44 ± 2.60	1.33 ± 0.50
	7	14.71 ± 1.95	7.61 ± 2.14	5.54 ± 1.85	1.78 ± 0.37
	14	16.09 ± 1.69	7.28 ± 1.00	5.33 ± 0.52	1.91 ± 0.35
	21	15.10 ± 3.81	7.43 ± 1.01	4.74 ± 1.04	1.40 ± 0.28
T + LC	5	14.54 ± 4.04	5.79 ± 1.90	4.87 ± 1.84	1.16 ± 0.50
	7	13.06 ± 1.52	7.32 ± 2.30	5.28 ± 1.89	1.70 ± 0.34
	14	15.97 ± 1.95	9.98 ± 1.87	7.12 ± 1.91	1.98 ± 0.42
	21	14.70 ± 3.26	7.31 ± 2.39	4.95 ± 1.42	1.50 ± 0.68
LC	5	9.63 ± 2.71	5.46 ± 1.59	4.37 ± 1.17	1.03 ± 0.31
	7	10.06 ± 1.67	6.57 ± 2.28	4.58 ± 1.43	1.86 ± 0.66
	14	10.14 ± 0.98	9.59 ± 1.83	7.21 ± 1.23	2.36 ± 0.61
	21	10.62 ± 1.93	8.31 ± 1.18	5.18 ± 0.83	1.62 ± 0.33
C	5	12.65 ± 4.53	4.44 ± 1.46	3.63 ± 1.33	1.16 ± 0.50
	7	10.99 ± 3.97	4.43 ± 1.46	3.51 ± 1.35	1.01 ± 0.55
	14	13.90 ± 4.24	7.92 ± 2.94	6.19 ± 2.10	1.42 ± 0.66
	21	13.36 ± 4.89	7.81 ± 3.03	5.72 ± 2.38	1.44 ± 0.64

**Table 3:** The percentage of mesenteric lymph nodes (MLN) B (CD19^+^) and T (CD3^+^, CD4^+^, CD8^+^) cells in groups of mice: infected with *T. spiralis* (Group T), infected with *T. spiralis* and receiving LC (Group T + LC), uninfected with *T. spiralis* and receiving LC (Group LC) and uninfected mice (control) (Group C). Mean values (*n* = 6) and standard deviations are presented. * *p* < 0.05, ** *p* < 0.01.

Group	Dpi	%CD19^+^ x ± SD	%CD3^+^ x ± SD	%CD4^+^ x ± SD	%CD8^+^ x ± SD	CD4^+^/8^+^ x ± SD
T	5	36.74 ± 6.13	58.97 ± 8.37	49.37 ± 7.46	9.34 ± 2.82	5.56 ± 1.46
	7	40.36 ± 4.27	52.38 ± 3.72	41.45 ± 3.01	11.44 ± 4.69	4.00 ± 1.18
	14	43.05 ± 5.45	49.28 ± 7.11	38.42 ± 6.38	11.61 ± 5.29	3.76 ± 1.45
	21	24.34 ± 14.12	72.88 ± 14.44	51.06 ± 11.95	18.32 ± 4.56	2.89 ± 0.93
T + LC	5	36.18 ± 4.18	57.71 ± 6.38	48.71 ± 5.02	11.21 ± 4.15	4.77 ± 1.46
	7	46.55 ± 7.70	48.26 ± 8.91	38.27 ± 6.98	9.61 ± 2.49	4.10 ± 0.68
	14	47.19 ± 11.08	47.33 ± 9.01	40.82 ± 4.46	8.30 ± 2.45	5.17 ± 1.06
	21	26.82 ± 7.20	68.97 ± 7.90	53.64 ± 10.13	17.08 ± 4.14	3.37 ± 1.24
LC	5	46.84 ± 8.51	36.99 ± 5.25	18.38 ± 2.54 **	14.56 ± 4.22	1.37 ± 0.54 *
	7	46.73 ± 5.35	51.11 ± 6.28	40.72 ± 5.46	10.66 ± 1.38	3.88 ± 0.78
	14	31.72 ± 7.07 *	59.31 ± 10.99	41.64 ± 10.74	11.31 ± 2.27	3.72 ± 0.78
	21	33.48 ± 6.26	63.10 ± 6.44	45.96 ± 4.18	20.83 ± 5.66	2.39 ± 0.87
C	5	44.52 ± 4.09	39.73 ± 7.40	34.50 ± 5.60	9.15 ± 2.71	3.90 ± 0.64
	7	38.83 ± 13.21	45.83 ± 15.07	38.13 ± 9.45	10.06 ± 3.11	3.89 ± 0.55
	14	46.43 ± 7.54	39.96 ± 7.38	34.80 ± 5.85	9.17 ± 2.72	3.92 ± 0.62
	21	31.43 ± 6.41	64.14 ± 9.64	46.44 ± 5.01	15.76 ± 4.11	3.07 ± 0.67

**Table 4:** The absolute count (cells × 10^6^) of mesenteric lymph nodes (MLN) lymphocytes B (CD19^+^) and T (CD3^+^, CD4^+^, CD8^+^) in groups of mice: infected with *T. spiralis* (Group T), infected with *T. spiralis* and receiving LC (Group T + LC), uninfected with *T. spiralis* and receiving LC (Group LC) and uninfected mice (control) (Group C). Mean values (*n* = 6) and standard deviations are presented. * *p* < 0.05, ** *p* < 0.01.

Group	Dpi	CD19^+^ x ± SD	CD3^+^ x ± SD	CD4^+^ x ± SD	CD8^+^ x ± SD
T	5	8.20 ± 1.51	13.29 ± 2.94	10.99 ± 1.77	2.13 ± 0.84
	7	13.12 ± 3.72	16.90 ± 3.95	13.28 ± 2.58	3.69 ± 1.83
	14	8.71 ± 2.53	10.66 ± 4.85	7.86 ± 2.91	2.60 ± 1.88
	21	3.66 ± 1.54	12.61 ± 6.88	9.01 ± 5.55	3.04 ± 1.48
T + LC	5	10.23 ± 2.53	16.47 ± 4.58	13.73 ± 3.02	3.34 ± 1.89
	7	18.35 ± 3.45 *	20.22 ± 8.88	15.78 ± 5.78	4.00 ± 1.62
	14	10.80 ± 4.51	10.24 ± 1.35	8.96 ± 1.57	1.76 ± 0.29
	21	8.64 ± 5.46	22.26 ± 13.73	16.08 ± 6.86	6.09 ± 4.94
LC	5	4.98 ± 2.54	3.69 ± 1.41	1.96 ± 1.02 **	1.36 ± 0.28
	7	6.96 ± 1.93	7.61 ± 2.23	6.21 ± 2.32	1.56 ± 0.29
	14	6.37 ± 0.93	12.33 ± 3.94	8.74 ± 3.56	2.34 ± 0.66
	21	6.46 ± 2.78	11.62 ± 2.84	8.35 ± 2.38	4.11 ± 1.42
C	4	7.14 ± 2.48	6.28 ± 1.88	5.56 ± 1.99	1.47 ± 0.64
	7	6.06 ± 3.24	6.53 ± 1.73	5.58 ± 1.72	1.47 ± 0.56
	14	8.04 ± 4.06	6.67 ± 2.44	5.97 ± 2.63	1.57 ± 0.79
	21	6.82 ± 2.28	14.30 ± 5.25	10.22 ± 3.22	3.64 ± 2.04

**Table 5:** Percentage of B (CD19^+^) and T (CD3^+^, CD4^+^ and CD8^+^) lymphocytes in murine peripheral blood (*n* = 6; x ± SD) *p* < 0.05 *, *p* < 0.01 **.

Group		Dpi	%CD19^+^ x ± SD	%CD3^+^ x ± SD	%CD4^+^ x ± SD	%CD8^+^ x ± SD	CD4^+^/8^+^ x ± SD
T		5	31.44 ± 6.98	49.89 ± 7.95	40.18 ± 9.18	9.93 ± 1.17	4.11 ± 1.09
		7	33.79 ± 2.93	51.92 ± 3.76	40.90 ± 7.07	10.67 ± 0.68	3.83 ± 0.62
		14	34.49 ± 4.52	44.89 ± 7.12	37.35 ± 4.38	10.14 ± 1.20	3.75 ± 0.79
		21	37.01 ± 5.36	40.89 ± 3.05	34.91 ± 3.52	11.65 ± 2.17	3.10 ± 0.70
T + LC		5	30.50 ± 5.84	51.33 ± 7.68	44.46 ± 6.94	10.96 ± 1.09	4.05 ± 0.38
		7	31.79 ± 5.66	53.07 ± 4.43	46.37 ± 4.78	10.39 ± 0.79	4.49 ± 0.62
		14	28.93 ± 6.24	52.26 ± 9.14	41.44 ± 8.36	11.42 ± 1.00 *	3.66 ± 0.84
		21	29.89 ± 4.04 *	42.85 ± 4.53	38.66 ± 3.99	9.90 ± 0.89	3.95 ± 0.66
LC	5		32.54 ± 5.94	53.35 ± 4.41	45.91 ± 4.64	10.29 ± 1.04	4.48 ± 0.43 *
	7		30.56 ± 3.58 **	54.31 ± 3.09 **	46.82 ± 2.67	11.67 ± 1.08 *	4.03 ± 0.28
	14		25.17 ± 3.63	60.83 ± 6.28	49.16 ± 3.78	13.24 ± 2.35	3.81 ± 0.71
	21		23.95 ± 5.40 *	55.88 ± 4.89 *	47.20 ± 4.32	12.70 ± 1.69 *	3.77 ± 0.54
C	5		28.93 ± 3.39	51.56 ± 4.15	43.01 ± 5.18	10.67 ± 0.64	4.03 ± 0.45
	7		25.87 ± 1.35	58.96 ± 2.76	48.17 ± 4.88	14.67 ± 3.70	3.51 ± 1.09
	14		24.57 ± 3.24	54.72 ± 4.83	43.84 ± 5.00	12.34 ± 2.36	3.70 ± 0.94
	21		28.31 ± 1.36	51.35 ± 1.61	44.57 ± 2.45	10.94 ± 1.20	4.12 ± 0.49

**Table 6:** Absolute count (cells × 10^6^) of B (CD19^+^) and T (CD3^+^, CD4^+^, CD8^+^) lymphocytes in murine peripheral blood. Mean values (*n* = 6) and standard deviations are presented. * *p* < 0.05, ** *p* < 0.01.

Group	Dpi	CD19^+^ x ± SD	CD3^+^ x ± SD	CD4^+^ x ± SD	CD8^+^ x ± SD
T	5	2.50 ± 0.65	4.02 ± 1.10	3.23 ± 1.01	0.80 ± 0.21
	7	2.82 ± 0.89	4.25 ± 1.10	3.34 ± 0.97	0.89 ± 0.26
	14	2.42 ± 0.55	3.10 ± 0.44	2.58 ± 0.31	0.70 ± 0.11
	21	5.65 ± 0.86	6.38 ± 1.51	5.47 ± 1.43	1.79 ± 0.39
T + LC	5	2.53 ± 0.95	4.09 ± 0.82	3.59 ± 1.05	0.89 ± 0.26
	7	1.95 ± 0.85	3.16 ± 0.87	2.76 ± 0.77	0.62 ± 0.19 *
	14	2.36 ± 0.86	4.24 ± 1.41 *	3.37 ± 1.20	0.92 ± 0.21 *
	21	2.91 ± 0.71 **	4.14 ± 0.81 **	3.73 ± 0.70*	0.96 ± 0.22 **
LC	5	3.03 ± 1.09	4.84 ± 0.82	4.15 ± 0.68	0.93 ± 0.17
	7	2.61 ± 0.67	4.60 ± 0.99 *	3.96 ± 0.82 *	0.98 ± 0.16 **
	14	2.47 ± 0.67	6.10 ± 2.06	4.92 ± 1.54	1.31 ± 0.45
	21	2.79 ± 0.51	6.66 ± 1.27 *	5.58 ± 0.73 *	1.51 ± 0.34 *
C	5	2.54 ± 0.74	4.45 ± 0.86	3.70 ± 0.74	0.92 ± 0.20
	7	2.92 ± 0.58	6.72 ± 1.68	5.52 ± 1.56	1.63 ± 0.41
	14	2.72 ± 0.41	6.12 ± 1.32	4.89 ± 0.97	1.41 ± 0.47
	21	2.88 ± 0.37	5.26 ± 0.91	4.55 ± 0.67	1.12 ± 0.24

**Table 7:** Proliferative response of splenocytes to ConA. Mean values (*n* = 6) and standard deviations are presented. Data with different superscript letters denote significant difference. a, b: *p* < 0.05; A, B: *p* < 0.01.

Dpi	Con A (µg/mL)	Proliferation Index			
		Group T x ± SD	Group T + LC x ± SD	Group LC x ± SD	Group C x ± SD
5	0.9	6.06 ± 0.73	6.61 ± 2.03	6.89 ± 1.34	7.01 ± 1.58
	0.45	2.79 ± 0.24	3.85 ± 0.64	3.37 ± 1.03	3.10 ± 0.73
	0.225	1.60 ± 0.18	2.22 ± 0.20	1.71 ± 0.41	1.93 ± 0.47
7	0.9	3.56 ± 0.85 ^a^	6.79 ± 0.71 ^b^	4.51 ± 1.31	6.09 ± 2.44
	0.45	3.46 ± 1.11	4.56 ± 1.27 ^A^	2.07 ± 0.58 ^B^	2.91 ± 0.92
	0.225	1.98 ± 0.61	2.50 ± 0.60 ^A^	1.31 ± 0.23 ^B^	1.13 ± 0.56 ^B^
14	0.9	3.71 ± 0.49	2.75 ± 0.57	5.12 ± 1.21	6.91 ± 2.24
	0.45	3.12 ± 0.40	2.44 ± 0.46	2.23 ± 0.83	4.72 ± 1.52
	0.225	1.87 ± 0.46	1.74 ± 0.29	1.64 ± 0.63	2.63 ± 0.41
21	0.9	2.55 ± 0.56	2.48 ± 1.04	4.75 ± 1.56	6.19 ± 2.19
	0.45	2.41 ± 0.81	2.46 ± 1.13	2.76 ± 0.94	4.10 ± 1.68
	0.225	1.44 ± 0.44	0.75 ± 0.25	0.83 ± 0.12	1.43 ± 0.61

**Table 8:** Blood parameters in the mice infected with *T. spiralis* (Group T) and infected with *T. spiralis* and receiving LC extract (Group T + LC). Mean values (*n* = 6) and standard deviations are presented. * *p* < 0.05, ** *p* < 0.01.

Parameter	5 Dpi		7 Dpi		14 Dpi		21 Dpi	
	Group T x ± SD	Group T + LC x ± SD	Group T x ± SD	Group T + LC x ± SD	Group T x ± SD	Group T + LC x ± SD	Group T x ± SD	Group T + LC x ± SD
WBC (10^3^/µL)	10.10 ± 1.56	11.35 ± 3.16	12.43 ± 3.74	9.37 ± 2.22	10.23 ± 2.03	11.03 ± 2.38	20.97 ± 3.14	16.12 ± 4.23 *
LYM%	73.63 ± 4.71	72.37 ± 7.00	64.45 ± 4.78	66.52 ± 3.82	70.02 ± 5.67	67.28 ± 5.24	70.60 ± 6.40	63.32 ± 13.02
MID%	10.52 ± 2.11	11.22 ± 2.81	14.70 ± 1.95	13.78 ± 1.75	13.32 ± 2.57	14.18 ± 2.81	13.08 ± 2.15	14.20 ± 6.35
GRAN%	15.85 ± 4.02	16.42 ± 6.93	20.85 ± 3.07	19.70 ± 3.36	16.67 ± 4.45	18.53 ± 2.78	16.32 ± 4.55	22.48 ± 9.16
LIMF (10^3^/µL)	7.50 ± 1.42	8.08 ± 1.80	8.02 ± 2.43	6.30 ± 1.78	7.13 ± 1.37	7.35 ± 0.93	14.92 ± 3.23	9.88 ± 2.11 **
MID (10^3^/µL)	1.08 ± 0.31	1.28 ± 0.52	1.87 ± 0.59	1.28 ± 0.23 *	1.35 ± 0.30	1.62 ± 0.74	2.68 ± 0.19	2.37 ± 1.35
GRAN (10^3^/µL)	1.52 ± 0.26	1.98 ± 1.28	2.55 ± 0.90	1.78 ± 0.39	1.75 ± 0.69	2.07 ± 0.78	3.37 ± 0.72	3.87 ± 2.52
RBC (10^6^/µL)	8.67 ± 0.52	7.81 ± 1.35	8.01 ± 0.39	7.54 ± 1.30	7.40 ± 0.45	7.35 ± 0.52	9.28 ± 0.23	8.46 ± 0.38 **
HGB (g/dL)	16.32 ± 0.90	14.98 ± 1.77	15.60 ± 0.80	13.87 ± 3.03	14.10 ± 0.90	13.53 ± 1.15	18.00 ± 0.51	16.97 ± 0.99 *
HCT (%)	53.97 ± 3.46	51.63 ± 6.47	52.22 ± 3.15	45.55 ± 8.13	47.03 ± 1.27	47.10 ± 2.98	59.30 ± 1.45	57.12 ± 4.52
MCV (fL)	62.33 ± 2.51	67.07 ± 5.56	65.43 ± 5.41	60.40 ± 1.25	63.77 ± 2.67	64.22 ± 1.73	63.98 ± 2.03	67.50 ± 2.58 *
MCH (pg)	18.78 ± 0.43	19.38 ± 1.96	19.45 ± 0.72	18.20 ± 0.97 *	19.02 ± 0.80	18.37 ± 0.70	19.35 ± 0.56	20.02 ± 0.60
MCHC (g/dL)	30.22 ± 0.70	29.03 ± 1.14	29.88 ± 1.99	30.20 ± 1.55	29.92 ± 1.39	28.67 ± 1.22	30.30 ± 1.13	29.70 ± 1.12
RDW-SD (fL)	26.62 ± 3.03	32.80 ± 10.02	33.75 ± 7.40	24.77 ± 1.91 *	28.78 ± 3.67	30.67 ± 3.66	29.38 ± 2.98	34.70 ± 5.33
RDW-CD (%)	14.48 ± 1.19	16.77 ± 3.79	17.57 ± 2.80	13.87 ± 1.02 *	15.40 ± 1.48	16.27 ± 1.68	15.70 ± 1.27	17.67 ± 2.20
PLT (10^3^/µL)	663.83 ± 110.0	595.17 ± 292.3	668.33 ± 246.8	616.00 ± 59.7	648.33 ± 78.7	731.00 ± 122.5	735.33 ± 99.7	694.50 ± 112.3
MPV (fL)	8.00 ± 1.04	7.43 ± 0.76	7.80 ± 1.15	7.65 ± 0.87	6.93 ± 0.18	6.90 ± 0.24	7.78 ± 0.95	7.25 ± 0.41
PDW (%)	8.55 ± 1.01	7.87 ± 0.08	8.17 ± 0.76	8.47 ± 0.88	8.03 ± 0.22	8.47 ± 0.72	8.13 ± 0.67	8.38 ± 0.62
PCT (%)	0.53 ± 0.15	0.45 ± 0.28	0.54 ± 0.31	0.47 ± 0.09	0.44 ± 0.04	0.50 ± 0.08	0.57 ± 0.13	0.50 ± 0.09
*p*-LCR (%)	5.42 ± 8.39	3.72 ± 9.10	3.63 ± 8.90	3.43 ± 6.76	0.00 ± 0.00	1.07 ± 1.67	3.02 ± 7.39	1.12 ± 2.74

**Table 9:** Blood parameters in uninfected mice (control) (Group C) and uninfected mice receiving LC extract (Group LC). Mean values (*n* = 6) and standard deviations are presented. * *p* < 0.05, ** *p* < 0.01.

Parameter	5 Day		7 Day		14 Day		21 Day	
	Group LCx ± SD	Group C x ± SD	Group LC x ± SD	Group C x ± SD	Group LC x ± SD	Group C x ± SD	Group LC x ± SD	Group C x ± SD
WBC (10^3^/µL)	15.57 ± 4.50	11.98 ± 2.13	11.92 ± 1.95 **	17.95 ± 3.43	13.35 ± 1.62	15.17 ± 3.75	16.12 ± 4.23	13.20 ± 2.22
LYM%	60.93 ± 12.58 *	74.62 ± 4.96	71.45 ± 5.85	63.80 ± 11.68	83.00 ± 1.73 *	71.38 ± 10.21	63.32 ± 13.02 *	79.1 ± 3.58
MID%	11.22 ± 4.46	9.98 ± 1.74	12.70 ± 2.59	11.85 ± 4.96	7.95 ± 1.27	9.65 ± 2.89	14.20 ± 6.35	8.63 ± 0.69
GRAN%	27.85 ± 13.03 *	15.40 ± 3.62	15.85 ± 3.87	24.35 ± 10.39	9.05 ± 0.99 *	18.97 ± 8.98	22.48 ± 9.16 *	12.15 ± 2.72
LIMF (10^3^/µL)	9.15 ± 2.11	8.97 ± 1.65	8.52 ± 1.46 *	11.33 ± 2.42	11.10 ± 1.37	10.60 ± 1.85	9.88 ± 2.11	10.43 ± 1.65
MID (10^3^/µL)	1.80 ± 1.21	1.18 ± 0.22	1.48 ± 0.40	2.07 ± 0.62	1.07 ± 0.19	1.53 ± 0.81	2.37 ± 1.35	1.17 ± 0.23
GRAN (10^3^/µL)	4.67 ± 3.11	1.83 ± 0.60	1.92 ± 0.62 *	4.55 ± 2.69	1.18 ± 0.25 *	3.03 ± 1.89	3.87 ± 2.52	1.60 ± 0.54
RBC (10^6^/µL)	8.37 ± 0.84	8.03 ± 1.03	8.03 ± 0.55 *	8.82 ± 0.32	8.86 ± 0.33 **	8.25 ± 0.19	8.46 ± 0.38	8.54 ± 0.15
HGB (g/dL)	15.58 ± 1.43	15.85 ± 1.19	15.30 ± 0.73 **	17.23 ± 0.69	16.75 ± 0.60 **	15.53 ± 0.26	16.97 ± 0.99	16.08 ± 0.43
HCT (%)	54.52 ± 4.51	52.52 ± 4.28	51.43 ± 2.87 **	56.87 ± 2.53	55.57 ± 2.91 **	51.42 ± 1.12	57.12 ± 4.52	56.23 ± 1.78
MCV (fL)	65.25 ± 1.73	65.97 ± 4.03	64.13 ± 1.71	64.53 ± 1.36	62.77 ± 1.24	60.67 ± 4.20	67.50 ± 2.58	65.90 ± 1.40
MCH (pg)	18.55 ± 0.58	19.85 ± 1.42	19.03 ± 0.60	19.48 ± 0.27	18.87 ± 0.36	18.77 ± 0.33	20.02 ± 0.60 **	18.78 ± 0.49
MCHC (g/dL)	28.55 ± 0.64 *	30.17 ± 1.15	29.72 ± 0.75	30.27 ± 0.75	30.12 ± 0.76	30.17 ± 0.35	29.70 ± 1.12	28.57 ± 1.09
RDW-SD (fL)	30.67 ± 3.25	32.50 ± 8.64	28.80 ± 2.57	28.18 ± 1.39	27.25 ± 1.52	27.58 ± 0.78	34.70 ± 5.33	29.73 ± 2.32
RDW-CD (%)	16.12 ± 1.43	16.82 ± 3.65	15.32 ± 1.11	14.95 ± 0.61	14.75 ± 0.70	14.98 ± 0.30	17.67 ± 2.20	15.48 ± 1.00
PLT (10^3^/µL)	498.17 ± 43.9	514.67 ± 93.6	543.50 ± 41.0	532.17 ± 81.4	638.00 ± 172.7	537.00 ± 74.8	694.5 ± 112.3 **	458.5 ± 72.9
MPV (fL)	7.52 ± 1.00	7.30 ± 0.18	7.60 ± 1.33	7.37 ± 0.16	7.50 ± 0.89	7.18 ± 0.04	7.25 ± 0.41	7.30 ± 0.18
PDW (%)	8.28 ± 0.72	8.13 ± 0.55	8.20 ± 0.73	7.90 ± 0.00	8.20 ± 0.73	7.90 ± 0.00	8.38 ± 0.62	7.85 ± 0.20
PCT (%)	0.37 ± 0.04	0.37 ± 0.06	0.41 ± 0.03	0.39 ± 0.06	0.49 ± 0.20	0.38 ± 0.05	0.5 ± 0.09 **	0.33 ± 0.05
P-LCR (%)	2.68 ± 6.57	0.38 ± 0.94	3.70 ± 9.06	0.00 ± 0.00	2.8 ± 6.90	0.00 ± 0.00	1.12 ± 2.74	0.00 ± 0.00

**Table 10:** Effect of LC on the number of *Trichinella spiralis* adults in the intestine and the number of larvae in the muscles of the infected mice.

	Dpi	Group T		Group T + LC	
		$\bar{x}$	SD	$\bar{x}$	SD
No. of adults of T. spiralis	5	60	36.23	79	29.43
	7	45.8	25.96	55.33	34.62
	14	0	0	4.3	6.7
	21	0	0	0	0
No. of muscle larvae of T. spiralis	60	23,667	12,162	24,900	12,348

**Table 11:** Identification and content (mg/g dw) of main compounds of honeysuckle berry (LC) extracts by UPLC-ESI-qTOF-MS/MS and HPLC-PDA.

Compounds	*t*_R_	UV λ_max_ (nm)	[M − H]^−^/[M + H]^+^ (*m/z*)	Other Ions (*m/z*)	LC Extract
Iridoids					(mg/g dw)
Loganic acid	3.73	245	375	213/191/151/169	64.27 ± 2.68
Loganic acid 7-*O*-pentoside	4.53	245	507	375/345/213/169/151.	18.04 ± 0.90
7-epi-loganic acid 7-*O*-pentoside	5.12	245	507	357/327/195/151	28.12 ± 0.46
Sweroside	5.73	245	357	195/125 (403 [M − H + HCOOH]^−^)	109.52 ± 1.33
Loganin	5.73	245	389	227/209 (435 [M − H + HCOOH]^−^)	
Loganin 7-*O*-pentoside	6.15	245	521	389/227 (567 [M − H + HCOOH]^−^)	27.03 ± 0.90
Sum of iridoids					246.98
Anthocyanins					
Cy 3.5-digluc	3.89	513	611^+^	449/287	4.91 ± 0.21
Cy 3-gluc	4.74	514	449	287	120.73 ± 4.32
Cy 3-rut	5.22	517	595^+^	287	7.27 ± 0.26
Pn 3-rut	6.10	516	463^+^	301	2.56 ± 0.06
Pn 3-glc	6.36	519	609^+^	301	1.27 ± 0.03
Sum of anthocyanins					136.74
Phenolic acids					
3-*O*-caffeoylquinic acid	2.45	325	353	191	6.17 ± 0.83
5-*O*-caffeoylquinic acid	3.62	325	353	191	38.29 ± 2.03
Caffeoylquinic acid	5.02	325	353	191	9.29 ± 0.42
Dicaffeoylquinic acid	8.39	326	515	353/191	9.34 ± 0.52
Sum of phenolic acids					63.09
Flavonols					
Quercetin *O*-vicianoside	6.74	353	595	301	1.37 ± 0.08
Quercetin 3-*O*-rutinoside	7.07	352	609	301	2.64 ± 0.15
Quercetin *O*-rhamoniside-*O*-hexoside	7.32	352	609	463/301	13.06 ± 0.21
Quercetin 3-*O*-glucoside	7.49	352	463	301	2.43 ± 0.03
Sum of flavonols					19.50

## Data Availability

Not available.

## Sample Availability

Not available.
